# Serrapeptase impairs biofilm, wall, and phospho-homeostasis of resistant and susceptible *Staphylococcus aureus*

**DOI:** 10.1007/s00253-022-12356-5

**Published:** 2023-01-13

**Authors:** Georgios Katsipis, Anastasia A. Pantazaki

**Affiliations:** Laboratory of Biochemistry, Department of Chemistry, Aristotle University of Thessaloniki, 54124 Thessaloniki, Greece

**Keywords:** *S. aureus*, MRSA, Biofilm, Serrapeptase, Alkaline phosphatases, DING

## Abstract

**Abstract:**

*Staphylococcus aureus* biofilms are implicated in hospital infections due to elevated antibiotic and host immune system resistance. Molecular components of cell wall including amyloid proteins, peptidoglycans (PGs), and lipoteichoic acid (LTA) are crucial for biofilm formation and tolerance of methicillin-resistant *S. aureus* (MRSA). Significance of alkaline phosphatases (ALPs) for biofilm formation has been recorded. Serrapeptase (SPT), a protease of *Serratia marcescens*, possesses antimicrobial properties similar or superior to those of many antibiotics. In the present study, SPT anti-biofilm activity was demonstrated against *S. aureus* (ATCC 25923, methicillin-susceptible strain, methicillin-susceptible *S. aureus* (MSSA)) and MRSA (ST80), with IC_50_ values of 0.67 μg/mL and 7.70 μg/mL, respectively. SPT affected bacterial viability, causing a maximum inhibition of − 46% and − 27%, respectively. Decreased PGs content at [SPT] ≥ 0.5 μg/mL and ≥ 8 μg/mL was verified for MSSA and MRSA, respectively. In MSSA, LTA levels decreased significantly (up to − 40%) at lower SPT doses but increased at the highest dose of 2 μg/mL, a counter to spectacularly increased cellular and secreted LTA levels in MRSA. SPT also reduced amyloids of both strains. Additionally, intracellular ALP activity decreased in both MSSA and MRSA (up to − 85% and − 89%, respectively), while extracellular activity increased up to + 482% in MSSA and + 267% in MRSA. Altered levels of DING proteins, which are involved in phosphate metabolism, in SPT-treated bacteria, were also demonstrated here, implying impaired phosphorus homeostasis. The differential alterations in the studied molecular aspects underline the differences between MSSA and MRSA and offer new insights in the treatment of resistant bacterial biofilms.

**Key points:**

• *SPT inhibits biofilm formation in methicillin-resistant and methicillin-susceptible S. aureus.*

• *SPT treatment decreases bacterial viability, ALP activity, and cell wall composition.*

• *SPT-treated bacteria present altered levels of phosphate-related DING proteins.*

**Graphical Abstract:**

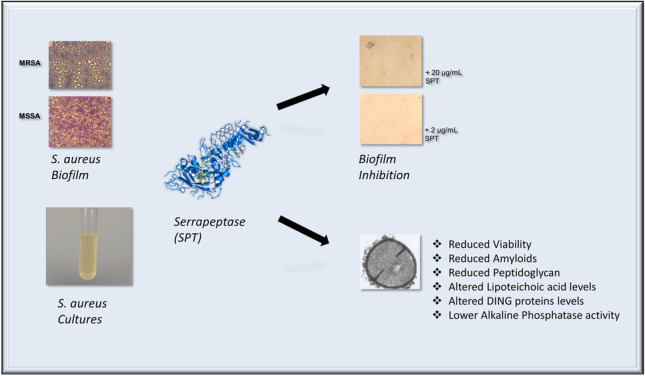

**Supplementary Information:**

The online version contains supplementary material available at 10.1007/s00253-022-12356-5.

## Introduction

*Staphylococcus aureus* (*S. aureus*), a Gram-positive bacterium that is frequently detected in the nasopharynx and the skin, is a central agent for hospital and community infections, including those of skin and soft tissues, bacteremia, osteomyelitis, infective endocarditis, pneumonia, and others (Bhattacharya et al. 2015; Guo et al. 2020). About one-fourth of healthy adults’ nasal cavities are positive for common methicillin-susceptible *S. aureus* (MSSA) (Choi et al. 2006; Munckhof et al. 2009; Ungureanu et al. 2017), while higher prevalence (even more than 50%) was recorded in individuals with extended exposure to microbial factors, e.g., athletes (Jiménez-Truque et al. 2016) or veterinary workers (Lee et al. 2018; Abdullahi et al. 2021). MSSA accounts for about one-fifth to three-fifths of total hospital infections (Ungureanu et al. 2017; Sapkota et al. 2019).

A fast-growing challenge for worldwide healthcare is the rise of resistant *S. aureus* strains that can counteract many of the most employed antibiotics like methicillin, and vancomycin, e.g., methicillin-resistant *S. aureus* (MRSA) and vancomycin-resistant *S. aureus* (Shariati et al. 2020). Of large interest is MRSA, a group of *S. aureus* strains first described in 1961, that is resistant to β-lactam antibiotics, e.g., penicillin, methicillin, and oxacillin (Lee et al. 2018; Turner et al. 2019). Estimates predict a prevalence of 2–53 million in southern Europe due to MRSA (Grundmann et al. 2006).

Chronic conditions linked to *S. aureus*, like implant-associated infections, wounds, osteomyelitis, cystic fibrosis lung infection, and endocarditis, were found to be highly dependent on biofilm formation (Bhattacharya et al. 2015). Biofilms are complex uni- or multi-microbial communities, encapsulated by an extracellular polymeric substance (EPS), in direct contact with biotic (tissues) or abiotic (implants, etc.) surfaces. Biofilm bacteria are highly resistant to host defense systems and may develop tolerance against antibiotics that naturally eliminate floating bacteria (planktonic) (Bhattacharya et al. 2015; Moormeier and Bayles 2017; Yin et al. 2019). *Staphylococcus* implant infections are confronted with long-term treatment of wide-spectrum antibiotics, and surgical removal (Oliveira et al. 2018). Sometimes, the employment of modified implants that possess antimicrobial properties is attempted, but these implants pose a threat for the development of more resistant strains. Thus, the implementation of specific and effective anti-biofilm treatment is of the vital need for present and future medicine (Oliveira et al. 2018; Ahmadabadi et al. 2020).

Though the formation of *S. aureus* biofilm has been largely studied, the molecular key players that are associated with this procedure are numerous and yet to be discovered in their entirety (Moormeier and Bayles 2017). Biofilm EPS is composed of a large variety of molecules: carbohydrates, extracellular DNA, and proteins. Biofilm channels, which are crucial for nourishment, remodeling, and virulence purposes, are also found in the structure (Periasamy et al. 2012). For the initial establishment of bacteria upon this matrix, a variety of cell wall–anchored *S. aureus* amyloid proteins is mobilized, namely surface protein G (Kuroda et al. 2008) and biofilm-associated protein (Taglialegna et al. 2016). As opposed to the toxic nature of amyloid-beta 42 peptide related to Alzheimer’s disease in humans and other animals (Findeis 2007), functional amyloids in bacteria are the building blocks of the fibers that initiate bacterial aggregation and binding to the EPS (Erskine et al. 2018). Cell wall components, including the abundant peptidoglycans (PGs) and lipoteichoic acids (LTAs), were also proven to be crucial for biofilm formation, maturation, and/or dispersion and are currently being studied as targets, for fighting biofilm infections (Szweda et al. 2012; Büttner et al. 2014; Ahn et al. 2018).

The activity of various enzymes like that of the proteases sortase A (Mazmanian et al. 1999) and autolysin A (Büttner et al. 2014) is recognized for biofilm formation. Alkaline phosphatases (ALPs) are pivotal bacterial enzymes for survival under phosphate-limited conditions (Gupta and Gupta 2021) and are lately recognized to be essential for biofilm formation; however, it is an enigmatic necessity in this process (Danikowski and Cheng 2018, 2019; Katsipis et al. 2021a). Phosphate is crucial for bacterial and biofilm physiology, as phosphate abundance or starvation alters bacterial metabolism, usually inducing the *Pho* regulon. *Pho* induction is largely dependent on the two-component system PhoBR, which functions through an ABC transporter involved in phosphorus transport (Gupta and Gupta 2021).

DING, a group of proteins known by a common N-terminus DINGGG homology, possesses a high capacity for phosphate binding. Crucial members of the DING family are phosphate metabolism–related proteins, including some ALPs and the PstS subunits of the ABC phosphate transporter. Though long studied, the physiological role of DING is largely putative and vague (Bernier 2013). In multi-drug-resistant strains of *Pseudomonas aeruginosa*, PstS-rich appendages of the DING family are characteristic of a highly virulent phenotype and are crucial for binding and infection of intestinal epithelial cells (Zaborina et al. 2008).

Serrapeptase (SPT), commonly also known as serratiopeptidase, is a metalloprotease first isolated from the Gram-positive bacterium *Serratia marcescens* (Miyata et al. 1970; Jadhav et al. 2020). Since its discovery, SPT is widely studied for its anti-inflammatory, analgesic (Tachibana et al. 1984; Nakamura et al. 2003; Al-Khateeb and Nusair 2008), anti-amyloid (Fadl et al. 2013; Metkar et al. 2017, 2020), and anti-biofilm (Longhi et al. 2008; Artini et al. 2011; Papa et al. 2013; Selan et al. 2015; Zapotoczna et al. 2015; Tsitsa et al. 2016; Selan et al. 2017) properties. SPT is tolerable and non-toxic for animal cells (Chopra et al. 2009; Papa et al. 2013; Selan et al. 2017), and several formulations (nanoparticles, liposomes, gels, etc.) have been designed to improve its delivery, bioavailability, and activity (KV et al. 2008; Shinde and Kanojiya 2014; Devlin et al. 2021). Though its anti-biofilm activity against *S. aureus* has been reported, the exact mechanism of action of SPT against *S. aureus* biofilm has not been elucidated yet (Artini et al. 2011; Selan et al. 2015).

In the present study, the effective anti-biofilm activity of SPT against MSSA (ATCC 25923) and MRSA ST80 is verified in a semi-quantitative manner and via microscopy. As far as we know, the anti-biofilm activity of SPT against MRSA ST80 has not been reported before. Furthermore, the inhibitory effect of SPT on bacterial viability is also indicated here, and the alteration in the physiological profile of several microbial components of the bacterial cell wall, namely PGs, amyloids, and LTA, is also demonstrated. Finally, to investigate the interrelation of phosphate homeostasis on biofilm formation, ALP activity and levels of DING proteins of SPT-treated bacteria have been evaluated. The present results should hopefully uncover a part of the mechanism concerning the anti-biofilm potential of SPT and would help bringing SPT again to the front as a safe, antimicrobial agent, to complement or even substitute some of the antibiotics employed in everyday medical practice.

## Materials and methods

### Chemicals and reagents

Serrapeptase capsules (60,000 IU per capsule) were purchased from Health Aid Ltd., UK. The whole content of a single capsule was suspended in the corresponding growth medium (1 capsule per 10 mL), vigorously vortexed for 2 min, and then filtered under sterile conditions (Minisart NY25, sterile 0.45-μM filters; Sartorius Stedim Biotech GmbH, Göttingen, Germany) to abort any non-dissolved enzyme and excipients. The preparation was then used without any further purification. The proteolytic activity of the preparations was tested with azocasein digestion (A-2765; Sigma-Aldrich, St. Louis, IL, USA), as previously described (Vélez-Gómez et al. 2019). The SPT content of the preparation was estimated with the Bradford-Bearden assay, as modified by Zor and Selinger (1996) (Bearden 1978). Tryptone (#403682), 3-(4,5-dimethylthiazol-2-yl)-2,5-diphenyltetrazolium bromide (MTT) (#A2231), *para*-nitrophenyl phosphate (pNPP) (#A1442), nitroblue tetrazolium (NBT) (#A1243), 5-bromo-4-chloro-3′-indolyl phosphate *p*-toluidine (BCIP) (#A1117), and other ingredients not already specified for culture media were purchased from PanReac AppliChem (Darmstadt, Germany). Soybean peptone (#70178), dimethyl sulfoxide (DMSO) (#D5879), crystal violet (CV) (#C0775), Congo red (CR) (#75768), Proteases Inhibitor Cocktail (#P-8849), ethylenediaminetetraacetic acid (EDTA), dithiothreitol (DTT), and phenylmethylsulfonyl fluoride (PMSF) were purchased from Sigma-Aldrich (St. Louis, MO, USA). Sterile double-distilled H_2_O was used throughout the experimental procedures.

### Bacterial strains and biofilm formation

MRSA (2679 ST80) was kindly provided from Dr. Marina Sagnou from the Institute of Biology of the Greek National Centre for Scientific Research “Demokritos” and was a clinical isolate from patients with urinary tract infection. *Staphylococcus aureus* strain ATCC® 25923™ was used as a wild-type strain in this study.

All growth media were autoclaved before use. Bacteria were stocked at − 20°C, in Luria-Bertani (LB) medium (% w/v: 1 tryptone, 0.5 NaCl, 0.5 yeast extract) containing 20% (v/v) filtered glycerol. Initial cultures were carried out with suspension of 100 μL of stock culture in 10 mL of LB medium and were grown overnight, at 37°C, in a shaking incubator. Biofilm formation was performed for both bacterial strains with Tryptic Soy Broth (TSB) medium (% w/v: 1.7 tryptone, 0.3 soybean peptone, 0.5 NaCl, 0.25 Κ_2_HPO_4_), supplemented with 1% (w/v) glucose. For biofilm formation, bacteria were grown for 24 h, at 37°C, in stative conditions. For the semi-quantification of biofilm formation, bacteria were grown in 96-well tissue culture plates (TCP), while for microscopical evaluation, biofilms were grown on glass slides. Biofilms were rinsed thoroughly with PBS and then stained with crystal violet (see the Supplementary Material for more details).

### Determination of bacterial viability by MTT assay

Though initially introduced for eukaryotic cells, the reduction of MTT by metabolically active bacteria is now demonstrated to be an effective method for estimating bacterial viability and metabolic activity (Wang et al. 2010; Grela et al. 2018). MRSA and MSSA were cultured in glass tubes sealed with a gauze cap filled with hydrophobic cotton, under biofilm conditions described previously, in the presence or absence of several SPT concentrations. Bacteria were vigorously mixed and harvested by centrifugation (5000 rpm for 10 min) and then washed and resuspended in PBS. The viability of the bacterial cells was determined with 0.5 mg/mL ΜΤΤ (final concentration), after incubation at 37°C for 30 min. A sample containing only the MTT substrate was also prepared as blank. Purple formazan crystals are received after centrifugation and dissolved with DMSO, and the absorbance was read at 570 nm, in a microplate reader. The bacterial viability was then expressed by setting the viability of the untreated bacteria at 100%. All results were normalized versus the rough bacterial density of the PBS-resuspended cells, as previously described (Wang et al. 2010).

### Determination of intracellular and extracellular ALP activities

Previous studies have proven the efficacy of measuring *S. aureus* ALP activity with the employment of the artificial phosphatase substrate, pNPP (Danikowski and Cheng 2019; Katsipis et al. 2021a). Bacteria of both strains were treated with SPT and collected as described in the previous section. To study the intracellular ALP activity, cells were resuspended in ALP buffer (100 mM NaCl, 100 mM Tris-HCl (pH 9.5), 5 mM MgCl_2_), containing 0.8 mM pNPP. A sample containing only the pNPP substrate was also prepared as a blank. The enzymatic reaction was performed at 37°C for 1 h, and then NaOH was added to terminate the reaction. Cells were discarded by centrifugation (13,500 rpm, 5 min), and the absorbance of the supernatant was read at 405 nm in a microplate reader. All results were normalized versus the rough bacterial density of the PBS-resuspended cells.

To study the extracellular ALP activity, growth media of the cultures were collected after removal of cells by centrifugation and, thereafter, condensed with lyophilization. The protein content of the concentrated media was estimated with the Bradford-Bearden assay, as modified by Zor and Selinger (1996) (Bearden 1978). Then, concentrated media were mixed with ALP buffer, containing 0.8 mM pNPP (final concentration) in the wells of a 96-well microplate, and incubated at 37°C for 1 h. Then, NaOH was added to stop the reaction and the absorbance was read at 405 nm, in a microplate reader.

### Estimation of amyloid and peptidoglycan content of bacterial surface

Amyloids on the bacterial surface were estimated with the CR assay, as previously described (Reichhardt et al. 2015). Additionally, CV is retained by the thick PGs layer of Gram-positive bacteria during Gram staining (Jones 2017), and thus, it could be employed as a measure for the estimation of the PGs content of bacterial cells. Briefly, bacterial suspension in PBS was mixed with CR at a final concentration of 10 μg/mL. Alternatively, CV was added to a final concentration of 10 μg/mL for the estimation of PGs content. Samples in the absence of bacteria were also prepared, to estimate the absorbance of the pure dye solutions. Bacteria were incubated at room temperature for 10 min and then removed by centrifugation. The absorbance of the supernatant for CR and CV was read at 500 nm and 570 nm, respectively. The dye retained from the bacteria was then inversely calculated, by subtracting the absorbance values from the ones received from samples containing only the dye solution, and the results were normalized versus the rough bacterial density of the PBS-resuspended cells.

### Lysis of whole bacterial cells

Bacteria from 10-mL cultures were collected as described and resuspended at 250 μL of a lysis buffer containing 100 mM Tris-HCl (pH 6.8), 10% (v/v) glycerol, 1% (w/v) sodium dodecyl sulfate (SDS), 2 mM EDTA, 20 mM DTT, and 2 mM PMSF. The bacterial suspension was freeze thawed (− 20°C/4°C) three subsequent times, with vigorous vortexing between cycles. Then, glass beads (0.5 mm, #Z250465, Sigma-Aldrich) were added to the centrifuge tubes at about 1/3 of the final mix volume, and the tubes were vortexed for 15 cycles of 20 s, with an interval of 1 min in ice. The bacterial lysate was then centrifuged at 4°C, to remove debris, and stored at − 20°C until further analysis. The protein content of the lysates was estimated with the BCA assay kit (#71285; Millipore, Darmstadt, Germany).

### Determination of DING proteins and LTA by dot blot analysis

For the detection of DING proteins, a rabbit polyclonal antibody produced against a 25–38 peptide containing the sequence DINGGGATLPQPLYC of phosphate ABC transporter periplasmic protein which binds phosphate (*Pseudomonas fluorescens* Pf-5; WP_011061052.1) (anti-DING) was prepared by GenScript (The Biology CRO) Company (Piscataway, NJ 08854, USA). For the detection of LTA, a mouse monoclonal antibody (#HM5018) against LTA (anti-LTA) was purchased from Hycult Biotech (Uden, The Netherlands). A goat anti-mouse IgG antibody conjugated with ALP (#SA00002-1) and a goat anti-rabbit IgG antibody conjugated with ALP (#SA00002-2) were employed as secondary antibodies and were purchased from Proteintech (Manchester, UK). All antibodies were diluted with PBS-0.05% (v/v) Tween 20 (PBS-T).

Dot blot analysis was employed for the semi-quantitative estimation of intracellular and extracellular contents of DING proteins or LTA, as previously described (Katsipis et al. 2021b). Briefly, 5 μL of either bacterial lysate or concentrated medium was spotted on a 0.45-μΜ nitrocellulose membrane (#71208, SERVA Electrophoresis GmbH) and air-dried. Blocking was performed with 5% (w/v) skimmed milk, at room temperature, and 1:1000 diluted anti-DING, or with 1:400 diluted anti-LTA, was added for overnight incubation, 4 °C. Then, the membrane was washed with PBS-T and the appropriate secondary antibody, diluted 1:2000, was added for 1.5 h, at room temperature. After washes, color development was performed for 30 min, 37 °C, with 0.5 mM NBT and 0.5 mM BCIP. To semi-quantify the received dots, ImageJ 1.49 (National Institutes of Health (NIH), USA) application was used. All results were normalized versus the total protein content.

### Statistical analyses

Statistical analyses and graph construction were performed with GraphPad Prism 8 (GraphPad Software, Inc.). All experiments were carried out at least in triplicates. In all provided graphs, bars represent mean values ± standard error of the mean (SEM). To examine differences between the untreated and treated groups, Brown-Forsythe and Welch one-way analysis of variance (ANOVA) tests were used, without correcting for multiple comparisons, and after verifying normality (Anderson-Darling, D'Agostino-Pearson omnibus, Shapiro-Wilk, and Kolmogorov-Smirnov). Inhibition at 50% of the untreated sample (IC_50_) and the corresponding 95% of confidence intervals (CI (95%)) were calculated based on the following equation: *log [SPT] vs. normalized inhibition (%), with variable slope*. Probable correlations were examined with Pearson’s analysis, determining *r* coefficients and two-tailed *p* values. The results of correlation analyses are summarized in Table 1. Statistically significant results were considered for *p* < 0.05. Notations for statistically significant differences between control (untreated) and treated samples are as follows: *p < 0.05, **p < 0.01, ***p < 0.001, and ****p < 0.0001.

**Table 1:** Correlation analysis of the biofilm formation of *S. aureus* with the factors studied. Pearson’s *R* and the corresponding *p* values are provided in the table, for all the studied factors and for both *S. aureus* strains, i.e., *S. aureus* ATCC 25923 and methicillin-resistant *S. aureus* ST80. Bolded numbers represent statistically significant results (*p* < 0.05). ND indicates factors that could not be determined during analysis and thus were not studied for correlation

Studied factor	*S. aureus* ATCC 25923 (MSSA)		Methicillin-resistant *S. aureus* ST80 (MRSA)	
	Pearson’s *R*	*p* value	Pearson’s *R*	*p* value
Viability	0.884	**0.0097**	0.850	**0.0340**
Amyloids	0.964	**0.0010**	− 0.480	0.1674
Peptidoglycan	0.924	**0.0042**	0.823	**0.0221**
Cellular LTA	− 0.710	0.0568	0.340	0.2884
Extracellular LTA	ND		− 0.990	**0.0006**
Cellular ALP activity	0.979	**0.0009**	0.914	**0.0054**
Extracellular ALP activity	− 0.859	**0.0042**	− 0.173	0.3716
Cellular DING	− 0.810	**0.0304**	0.770	0.0636
Extracellular DING	ND		− 0.975	**0.0024**

## Results

### SPT inhibits biofilm formation of MRSA and MSSA

**Fig. 1:**
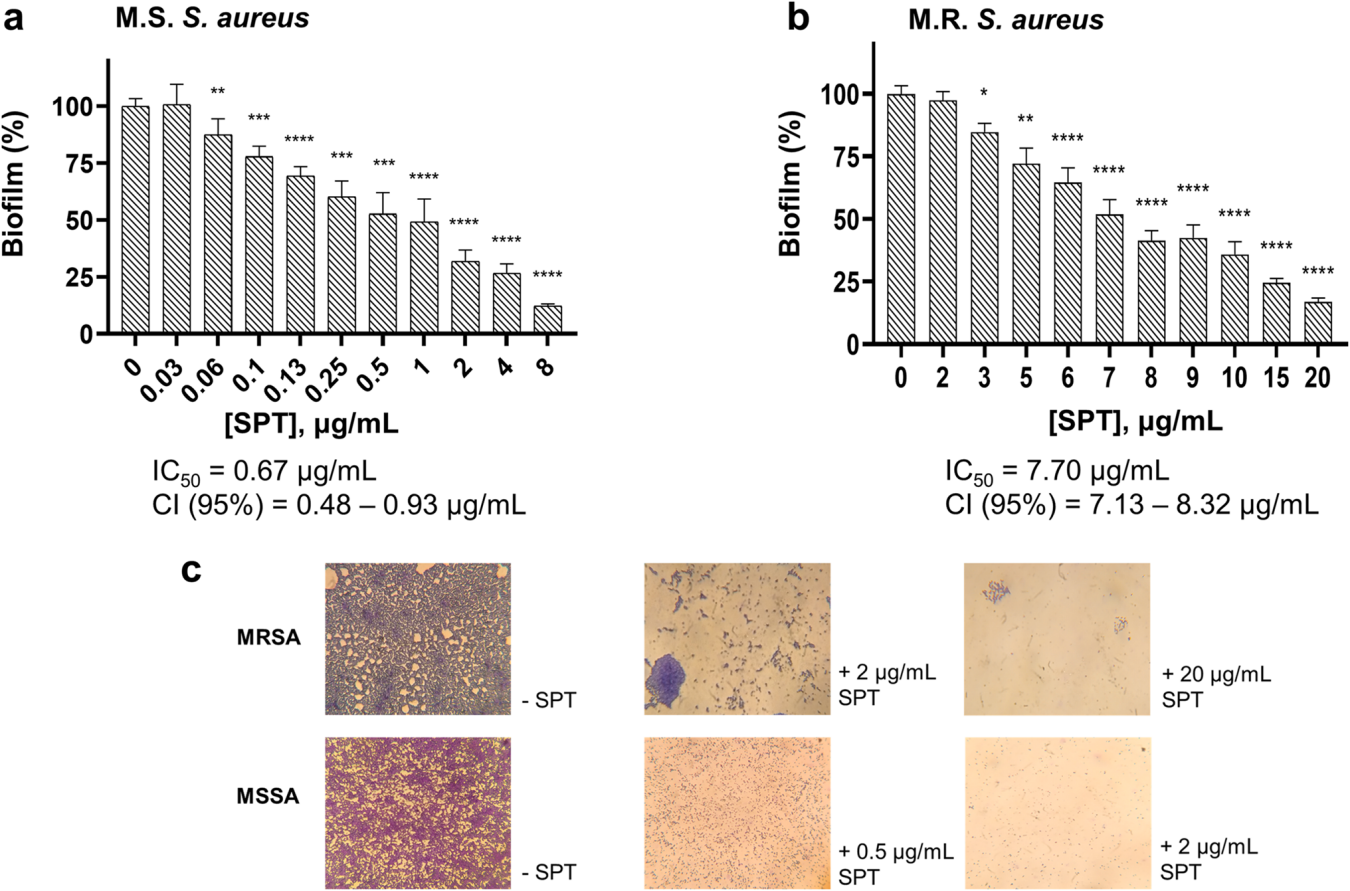
Inhibition of biofilm formation **a** of *S. aureus* ATCC 25922 (MSSA) and **b** of *S. aureus* ST80 (MRSA), by SPT. Bacteria were grown under stative conditions, in the presence or absence of several concentrations of STP (0.03–20 μg/mL) in 96-well TCPs. Bars represent mean values ± SEM from at least three independent experiments, with the value of the untreated bacterial culture (control) set at 100%. Brown-Forsythe tests and Welch ANOVA were employed for the statistical analysis. Notations for statistically significant differences between control (untreated) and treated samples: *p < 0.05; **p < 0.01; ***p < 0.001; ****p < 0.0001. **c** Biofilms formed on glass slides in the absence (−SPT) or presence (+SPT) of SPT and stained with CV. Images are taken from × 100 magnification under a light microscope

MSSA and MRSA were grown under biofilm conditions, with or without the addition of various SPT concentrations (ranging from 0.03 to 20 μg/mL). SPT effectively inhibited biofilm formation in both MSSA and MRSA strains, in a significant manner. For MRSA, statistically significant reductions in biofilm formation, when compared with control, were recorded at [SPT] > 2 μg/mL, while those for MSSA were recorded at [SPT] > 0.03 μg/mL (Fig. 1a, b). SPT was a more potent inhibitor for biofilm formation of MSSA, with an IC_50_ value calculated at 0.67 μg/mL (CI (95%) = 0.48–0.93 μg/mL), while for MRSA, the calculated IC_50_ value was 7.70 μg/mL (CI (95%) = 7.13–8.32 μg/mL). The maximum inhibition of biofilm formation for the studied concentrations was − 88% for MSSA at [SPT] = 8 μg/mL and − 83% for MRSA at [SPT] = 20 μg/mL.

To better observe the obstructing effect of SPT on *S. aureus* biofilm, bacterial biofilms were also grown and observed on glass slides. Representative photographs are provided in Fig. 1c. In the absence of SPT (−SPT), bacteria are grown in dense, organized micro-communities, immensely interacting with each other. Especially for MRSA, SPT treatment (+SPT) disrupts biofilm architecture, including water channels, as only scattered, small clusters of cells could be visualized. These observations clearly support the anti-biofilm efficacy of SPT.

### SPT reduces the viability of MRSA and MRSA

**Fig. 2:**
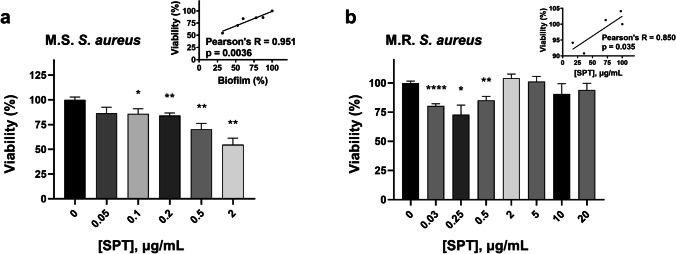
Effect of several concentrations of SPT on the viability **a** of *S. aureus* ATCC 25922 (MSSA) and **b** of *S. aureus* ST80 (MRSA). Bacteria were grown under biofilm conditions, collected after 24 h, and the viability has been determined with MTT assay. Bars represent mean values ± SEM from at least three independent experiments, with the value of the untreated bacterial culture (control) set at 100%. Brown-Forsythe tests and Welch ANOVA were employed for the statistical analysis. Notations for statistically significant differences between control (untreated) and treated samples: *p < 0.05; **p < 0.01; ***p < 0.001; ****p < 0.0001. Inset in **a**: correlation analysis of MSSA viability with biofilm formation. Inset in **b**: correlation analysis of MRSA viability with biofilm formation

For exploring the effect of SPT on bacterial viability and metabolic efficiency, the MTT test was employed. SPT treatment reduces the viability of MSSA for all studied concentrations of SPT employed; however, statistical significance was succeeded at [SPT] ≥ 0.1 μg/mL. Viability reduction was up to − 46% at the higher studied SPT dose of 2 μg/mL. Additionally, correlation analysis proved that the reduction in bacterial viability is strongly linked to a statistically significant reduction in biofilm formation, as it is provided in the inset of Fig. 2a.

SPT treatment of MRSA led to significant reductions of viability for concentrations of SPT below 0.5 μg/mL, with a maximum inhibition of − 27% demonstrated at [SPT] = 0.25 μg/mL. At higher SPT doses, bacterial viability was not found to differ from the untreated sample in a statistically significant manner. A less significant and weaker correlation of viability with biofilm formation was also demonstrated for MRSA and is provided in the inset of Fig. 2b. These results prove that SPT treatment can therefore impede proper active metabolism, especially on MSSA, and this effect could be implicated in biofilm formation.

### SPT treatment modifies the cell wall composition of MRSA and MSSA

**Fig. 3:**
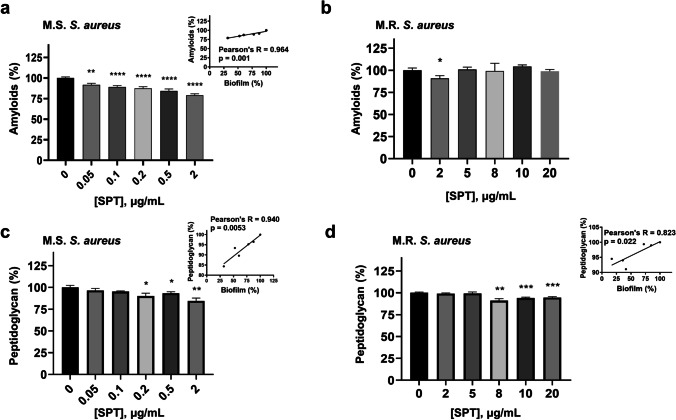
Effect of several concentrations of SPT on amyloids or peptidoglycan of **a**, **c** *S. aureus* ATCC 25922 (MSSA) and of **b**, **d** *S. aureus* ST80 (MRSA). Bacteria were grown under biofilm conditions, and their amyloid or peptidoglycan content has been determined inversely with retainment of Congo red or crystal violet, respectively. Bars represent mean values ± SEM from at least three independent experiments, with the value of the untreated bacterial culture (control) set at 100%. Brown-Forsythe tests and Welch ANOVA were employed for the statistical analysis. Notations for statistically significant differences between control (untreated) and treated samples: *p < 0.05; **p < 0.01; ***p < 0.001; ****p < 0.0001. Inset in **a**: correlation analysis of MSSA amyloids with biofilm formation. Inset in **c**: correlation analysis of MSSA peptidoglycan with biofilm formation. Inset in **d**: correlation analysis of MRSA peptidoglycan with biofilm formation

The possible effect of SPT on amyloid compounds and PGs was studied with two signature dyes, i.e., CR for amyloids and CV for PGs. SPT dose-dependently reduced the amyloid content of MSSA (Fig. 3a), with the observed alterations being strongly significant in a statistical manner. The maximal reduction for the employed SPT concentrations was − 21%, at [SPT] = 2 μg/mL. Statistical analysis proved that the amyloid content correlates in a strong, positive manner with biofilm formation (inset of Fig. 3a). Significantly reduced amyloid quantities for MRSA were only found also at 2 μg/mL of [SPT] (− 9%), but no significant alterations compared to control were demonstrated for the other studied concentrations of SPT (Fig. 3b). No significant correlation was proven between bacterial amyloids and biofilm formation in MRSA.

SPT treatment led to lower PGs content in both MSSA (Fig. 3c) and MRSA (Fig. 3d). Significant reductions of PGs were recorded for treatment of MSSA with [SPT] ≥ 0.5 μg/mL (up to − 16% at [SPT] = 2 μg/mL) and for treatment of MRSA with [SPT] ≥ 8 μg/mL (up to − 9% at [SPT] = 8 μg/mL). Correlation analysis demonstrated that for both MSSA and MRSA, the PGs content interrelates significantly, in a positive manner, with the biofilm formation (insets of Fig. 3c, d).

Finally, the levels of LTA at both cell lysate (intracellular LTA) and the culture media (extracellular LTA) were estimated with dot blot analysis. Treatment with SPT significantly impaired intracellular LTA levels (normalized OD in AU) in both MSSA and MRSA, but variably.

**Fig. 4:**
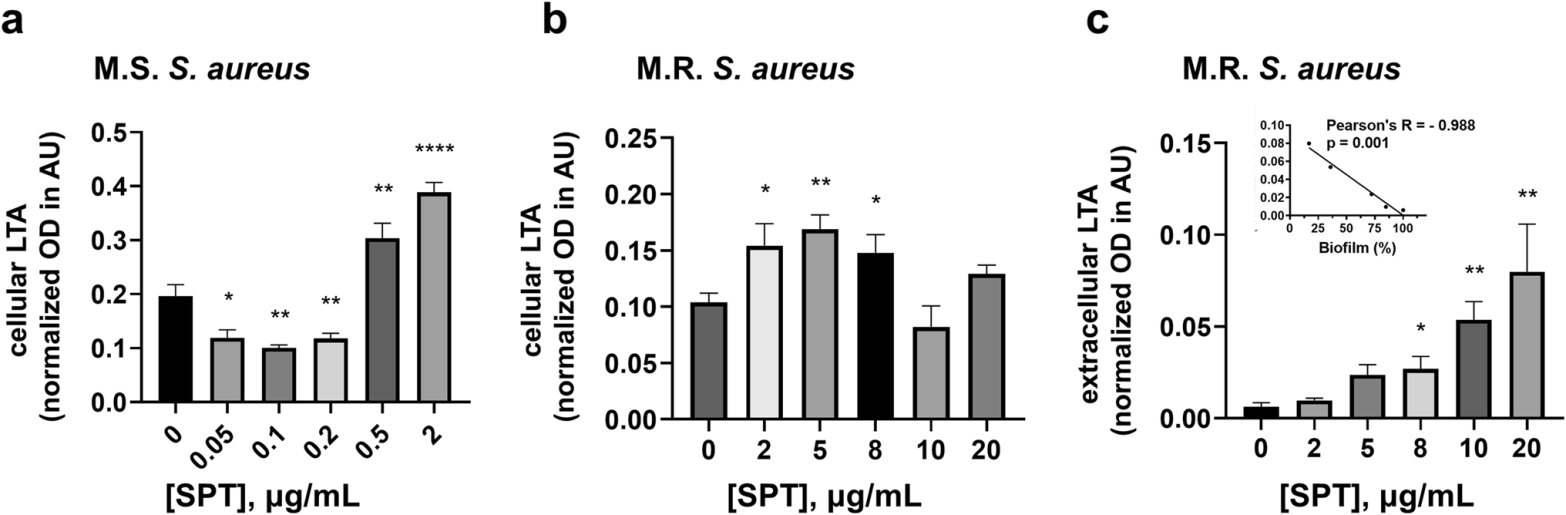
Levels of lipoteichoic acids (LTAs) after treatment with several concentrations of SPT. Intracellular levels of **a** *S. aureus* ATCC 25922 (MSSA) and **b** *S. aureus* ST80 (MRSA) and **c** extracellular levels at MRSA. Bacteria were grown under biofilm conditions and lysed by bead beating. LTA levels in cell lysates or concentrated media were determined with semi-quantitative dot blot analysis, employing a monoclonal anti-LTA. All results are normalized against the total sample protein. Bars represent mean values ± SEM from at least three independent experiments. Brown-Forsythe tests and Welch ANOVA were employed for the statistical analysis. Notations for statistically significant differences between control (untreated) and treated samples: *p < 0.05; **p < 0.01; ***p < 0.001; ****p < 0.0001. Inset in **c**: correlation analysis of LTA levels of MRSA with biofilm formation. Inhibition of MRSA biofilm strongly correlates inversely with the increase of extracellular LTA in a statistically significant manner

For SPT treatment ≤ 0.2 μg/mL, LTA of MSSA was found to decrease in a significant manner (up to − 40% at 0.1 μg/mL of SPT), but for [SPT] = 2 μg/mL, its levels increased spectacularly and significantly, up to + 114% (Fig. 4a). Adversely, SPT treatment leads to a strong, statistically significant increase of LTA levels in MRSA, at [SPT] ≤ 8 μg/mL (up to + 62.5%, at [SPT] = 5 μg/mL), while for higher studied concentrations of SPT, LTA levels do not significantly differ from the control (Fig. 4b).

LTA extracellular levels could not be detected at MSSA, while extracellular basal levels of LTA at MRSA were barely detectable, but significantly increased after SPT treatment, in a dose-dependent manner (up to + 1233%, at [SPT] = 20 μg/mL), as shown in Fig. 4c. The latter effect was found to strongly correlate, in an inverse, statistically significant manner with biofilm formation (inset at Fig. 4c), proving the release of LTA from MRSA cells is immensely linked with a reduced capability for biofilm formation. Collectively, the above results for both bacteria prove the ability of SPT to impair bacterial surface architecture.

### Biofilm inhibition by SPT is related to phosphate dyshomeostasis at MSSA and MRSA

**Fig. 5:**
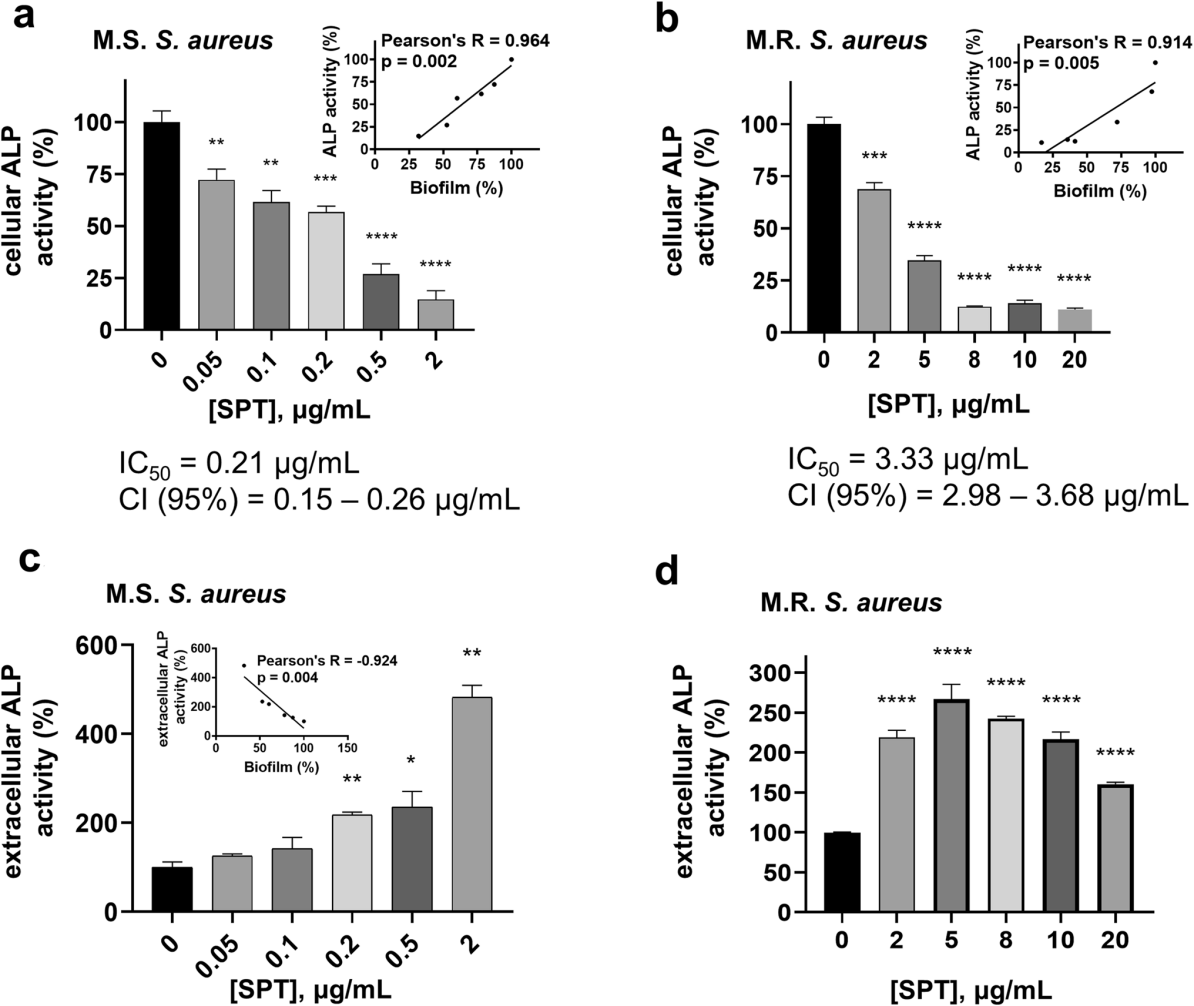
Effect of several concentrations of SPT on alkaline phosphatase (ALP) activity. **a** Intracellular and **c** extracellular activities from *S. aureus* ATCC 25922 (MSSA). **b** Intracellular and **d** extracellular activities from *S. aureus* ST80 (MRSA). Bacteria were grown under biofilm conditions. The activity of ALPs was estimated with the hydrolysis of the synthetic substrate pNPP. Bars represent mean values ± SEM from at least three independent experiments, with the value of the untreated bacterial culture (control) set at 100%. Brown-Forsythe tests and Welch ANOVA were employed for the statistical analysis. Notations for statistically significant differences between control (untreated) and treated samples: *p < 0.05; **p < 0.01; ***p < 0.001; ****p < 0.0001. Inset in **a**: correlation analysis of MSSA intracellular ALP activity against biofilm formation. Inset in **b**: correlation analysis of MRSA intracellular ALP activity with biofilm formation. Inset in **c**: correlation analysis of MSSA extracellular ALP activity with biofilm formation

Based on the previously proven significant interrelation of biofilm formation with ALPs (Danikowski and Cheng 2018, 2019; Katsipis et al. 2021a), the possible implication of their activity to the SPT-induced biofilm inhibition was studied here, employing the artificial substrate pNPP. Intracellular ALP activity was found to be significantly decreased, for all studied concentrations of SPT, in a dose-dependent manner, for both MSSA (Fig. 5a) and MRSA (Fig. 5b). The maximum inhibition of cellular ALPs for MSSA was − 85%, at [SPT] = 2 μg/mL, and that for MRSA was − 89%, at [SPT] = 20 μg/mL. As presented in the insets of Fig. 5a and b, ALP activity correlates strongly, in a positive and statistically significant manner, with biofilm formation for both bacteria.

ALP activity detected in the media of the bacterial cultures, which reflects the secreted/extracellular levels of ALPs, presented a much different profile. In both bacteria, extracellular ALP activity increased when compared to the control. For MSSA (Fig. 5c), extracellular/secreted ALP activity increased significantly for [SPT] ≥ 0.2 μg/mL, with maximum levels of + 482% at SPT treatment of 2 μg/mL, while for MRSA (Fig. 5d), a maximum increase of + 267% activity was found at 5 μg/mL of SPT treatment, while at higher concentrations of SPT, the increase was less strong, though still significant. Additionally, extracellular ALP activity correlates strongly, in a negative and statistically significant manner, with biofilm formation of MSSA (inset of Fig. 5c). Such a correlation was not statistically proven for MRSA. The abovementioned results underline the tight connection between ALP activity and biofilm formation for both *S. aureus* strains.

**Fig. 6:**
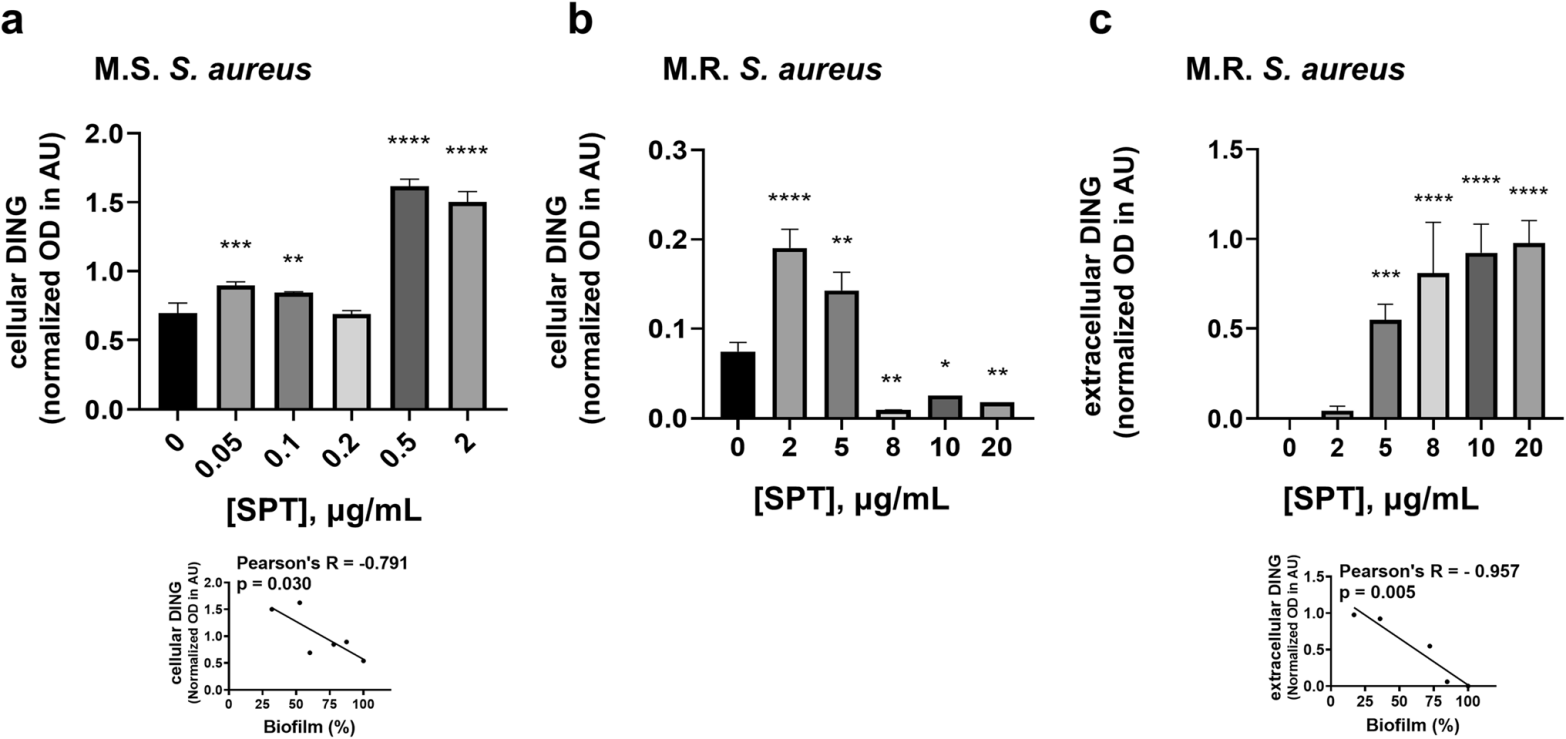
Levels of DING proteins after treatment with several concentrations of SPT. Intracellular levels **a** of *S. aureus* ATCC 25922 (MSSA) and **b** of *S. aureus* ST80 (MRSA) and **c** extracellular levels of MRSA. Bacteria were grown under biofilm conditions and lysed by bead beating. LTA levels in cell lysates or concentrated media were determined with semi-quantitative dot blot analysis, employing a polyclonal antibody produced against a 25–38 peptide containing the sequence DINGGGATLPQPLYC. Bars represent mean values ± SEM from at least three independent experiments. Brown-Forsythe tests and Welch ANOVA were employed for the statistical analysis. Notations for statistically significant differences between control (untreated) and treated samples: *p < 0.05; **p < 0.01; ***p < 0.001; ****p < 0.0001. Inset in **a**: correlation analysis of DING levels of MSSA with biofilm formation. Inset in **c**: correlation analysis of extracellular DING levels of MRSA with biofilm formation

Based on the results for ALP activity and on the notion that SPT treatment may impair phosphate homeostasis in *S. aureus*, DING, a family of proteins implicated in phosphate binding and uptake, was also analyzed here. Intracellular DING proteins were found significantly increased at MSSA, for all studied SPT concentrations, except for 0.2 μg/mL of SPT (Fig. 6a). A maximum increment of DING levels was found at [SPT] = 0.5 μg/mL and was + 133%, in comparison with the untreated sample. The levels of intracellular DING proteins were found to correlate inversely with biofilm formation (inset of Fig. 6a). On the other hand, intracellular DING levels at MRSA presented a somewhat “bipolar” behavior, with a strong, significant increase at [SPT] ≤ 5 μg/mL, followed by an abrupt, strong decrease at [SPT] ≥ 8 μg/mL (Fig. 6b). Maximum levels of intracellular DING in MRSA were + 157% when compared to control, at [SPT] = 2 μg/mL, and the minimum levels were − 87%, at [SPT] = 10 μg/mL.

Extracellular DING levels of MSSA and of the control sample of MRSA (Fig. 6c) were undetectable. Interestingly enough, extracellular DING levels of MRSA greatly increased, and in a significant manner, when bacteria were treated with SPT of concentrations ≥ 5 μg/mL. Due to the undetected levels of DING proteins in the media of untreated bacteria, no percentage of alteration in comparison with control can be provided. Extracellular DING levels increased in a dose-dependent manner, for all studied treatments of SPT on MRSA, and were found to correlate negatively with the biofilm formation (inset of Fig. 6c).

## Discussion

Colonizing the human body is followed by a long, strenuous, and most often lethal pursuit for germs. As a counter to the artificial, ideal conditions employed in laboratory environments, microbes in real life must cope with a plethora of adverse conditions, including nutrient deficiency, pH variances, and a full-frontal assault and repression from the host immune system. In that manner, biofilms are regarded as most certainly the best survival option and a widely distributed and successful mode of life (Yin et al. 2019). Bacterial biofilms are currently implicated in a variety of health issues, including tissue-associated, device-associated, and bloodstream or urinary tract infections (Srivastava and Bhargava 2016). Modern aspects of possible biofilm-related disorders also include persistent inflammatory reactions involved in autoimmune diseases (rheumatoid arthritis, inflammatory bowel disease, etc.) and neurodegenerative diseases (Alzheimer’s and Parkinson’s diseases) (Wolcott 2020; Miller et al. 2021). At the same time, antibiotic resistance is a fast-growing complication for modern medicine, linked with increased levels of morbidity and mortality, while biofilms are implicated in the gaining of multi-drug resistance (Frieri et al. 2017).

For all the aforementioned reasons, it is imperative to implement new medications as alternates to antibiotics against microbial biofilms. So, in the current study, the effect of SPT on *S. aureus* biofilms was studied. Given the emerging threat of antibiotic-resistant *S. aureus* (Shariati et al. 2020), an MRSA strain prevalent in South Europe and Greece, namely MRSA ST80, has been chosen (Drougka et al. 2014; Lee et al. 2018), alongside a susceptible strain, ATCC 25923. On that manner, this study addresses questions in two axes: (a) the anti-biofilm potency of SPT against *S. aureus* and the possible implication of antibiotic resistance on that, and (b) the molecular mechanisms underlying the different aspects of biofilm formation from the methicillin-resistant strains, with a focus on cell wall and phosphate metabolism.

SPT is a potent inhibitor against biofilm formation from both MRSA and MSSA, as proven by the TCP method and microscopic evaluation. BSA or trypsin was also studied here for the sake of comparison (Fig. S1) and did not present similar results, implying that SPT possesses a specific anti-biofilm activity, that is not attributed to an excess of protein or proteolytic potential at the growth medium. Additionally, the SPT-treated bacteria, especially in the case of MSSA, presented significantly less metabolic activity, as determined by the MTT viability assay, and that effect was correlated with the hindrance of biofilm formation. The toxic effect of SPT on *S. aureus* has been previously studied with the plate method on tryptone soy agar, followed by counting of colony-forming units (CFUs) (Hogan et al. 2017). The MTT assay employed here is based on the metabolic activity of bacteria (mainly oxidoreductive enzymes) and thus is giving some alternate insights into the inhibitory effect of SPT on bacterial viability (Grela et al. 2018). The increase in antibiotic susceptibility found by Hogan et al. (2017) could be attributed to an effect of SPT on bacterial metabolism, leading to greater sensitivity towards the antibiotic treatment.

The ability of SPT to block *S. aureus* biofilms has been described before for *S. aureus* ATCC 6538P (Artini et al. 2011; Papa et al. 2013; Selan et al. 2015); SH100 (Hogan et al. 2017); ATCC 25923, ATCC 12598, and MRSA ATCC BAA1556 (Papa et al. 2013); and USA300 JE2 (Hogan et al. 2017). However, as far as we know, inhibition of MRSA ST80 clone biofilm by SPT is reported here for the first time. ST80 is a growing threat to Greek and North European health systems, reaching a prevalence in Greece of over 73%, from 28%, among isolated MRSA strains, within a decade (Drougka et al. 2014). Additionally, MRSA ST80 is a dominant cause of tissue infections and is proven to successfully evade host immune system inflammatory reactions and thus being able to thrive and lead to long infections within communities and hospitals (Kolonitsiou et al. 2019). Thus, SPT empoyment could be proven promising to halt the spread of this resistant clone and its effects on healthcare system.

Interestingly, the current study presents a significant difference in the inhibitory activity of SPT against biofilm formation by MSSA compared to MRSA. In detail, 11.5 times higher SPT concentration is required to achieve half inhibition (IC_50_) in MRSA compared to MSSA. Since the main difference between these two *S. aureus* strains lies in the multi-drug resistance of MRSA to antibiotics, these results could indicate a possible mechanism of action of SPT, which is significantly more active in non-resistant strains, and simultaneously, that MRSA strains have developed a defense system against anti-biofilm agents. A previous study demonstrated stronger biofilm formation capacity in MRSA in comparison with MSSA (Hosseini et al. 2020), and biofilm formation was previously correlated with increased multi-drug resistance in *S. aureus* clinical isolates (Kwon et al. 2008; Sun et al. 2018). However, several other studies did not find any significance between methicillin resistance and biofilm formation (Smith et al. 2008; Ghasemian et al. 2016; Senobar Tahaei et al. 2021), and thus, the methicillin-resistant phenotype is not necessarily connected to the different effects of SPT on biofilm formation.

Biofilm growth by MSSA is highly dependent on the *ica* operon, which encodes the basic biosynthetic enzymes for biofilm EPS formation. On the other hand, in MRSA strains, biofilm formation can occur without the need for *ica* induction (McCarthy et al. 2015). It, therefore, appears that biofilm EPS in MRSA can be formed by alternate mechanisms, most likely by the use of adhesins (O’Neill et al. 2007). SPT may affect one of the mechanisms of EPS formation related to the operon *ica*, or the bacteria, interaction/adhesion mechanisms to the EPS, and thus, the inhibitory effect upon MSSA is more pronounced.

Results of the current study seem to reflect SPT effects on the bacterial cell wall in both MSSA and MRSA, concerning the estimation of PGs content with CV staining. Reductions in PG content were found for both *S. aureus* strains, in positive correlation with the biofilm inhibition. It was previously demonstrated that PGs content is directly linked with the quantity of cell wall–anchored proteins (Kim et al. 2018) and that biofilm cells produce increased titers of proteins needed for cell attachment and PGs synthesis (Resch et al. 2006). Additionally, PGs hydrolysis and/or remodeling is actively engaged with biofilm formation (Szweda et al. 2012; Büttner et al. 2014), verifying the results found here. Hamamelitannin, a quorum-sensing inhibitor, was also found to inhibit biofilm formation in *S. aureus*, by simultaneously decreasing PGs and cell wall thickness, reflecting in strong downregulation of several genes related to PG synthesis in the process (Brackman et al. 2016). Anti-biofilm effect of tannic acid in *S. aureus* was also linked with PGs damage and loss of cell wall integrity (Dong et al. 2018). All these results imply that the PGs content of bacteria is crucial for effective biofilm formation, and it is to be determined whether SPT is impairing the cell wall directly or indirectly.

The anti-biofilm activity of SPT was initially assumed to be proteolytic (Artini et al. 2011); however, subsequent studies by the same research team showed that artificial mutations at the active site of SPT did not affect its inhibitory effect against biofilm formation in *S. aureus* ATCC 6538P. These results thus indicate that SPT activity is not due to direct proteolysis of some surface or other factors, but to some other unknown properties of SPT (Selan et al. 2015). However, SPT treatment negatively affects the presence of several surface proteins (autolysin, IgG-binding proteins, ssaA antigen, and sdrD protein). All these factors appear to be implicated in bacterial adherence, infection, and biofilm formation (Papa et al. 2013). Several surface cell wall–anchored proteins have been proven to contribute, in addition to the *ica* operon products, to the formation of *S. aureus* biofilms and, in particular, the ability of cells to bind to the extracellular matrix (Speziale et al. 2014), as well as to form planktonic aggregates (Haaber et al. 2012). Among them, *S. aureus* surface protein G and biofilm-associated protein have been shown to have amyloidogenic properties (Kuroda et al. 2008; Taglialegna et al. 2016). *S. aureus* strains that do not express *S. aureus* surface protein G protein cannot form cell aggregates (Formosa-Dague et al. 2016). In the current study, amyloid-forming proteins, as detected by the signature CR staining, were affected in both MSSA and MRSA after SPT treatment. The anti-amyloid potency of SPT has been demonstrated in several studies, by inhibiting insulin aggregation, both in vitro (Metkar et al. 2017; Venkataprasad et al. 2021) and in vivo (Metkar et al. 2017, 2020). In the present work, it has not been possible to determine whether the inhibitory action of SPT is due to direct proteolytic activity. However, given the employment of SPT from time zero of biofilm formation and based on the conclusions of Selan and her colleagues (2015), it is hypothesized that SPT may intervene indirectly in the initial stages of agglomeration, possibly through an alternate mechanistic pathway than proteolysis.

Among the cell wall constituents, LTA is regarded as a major component and is implicated in various physiological roles, including division, separation, host recognition, and biofilm formation. LTA can also be excreted during bacterial growth, participating in bacterial virulence through Toll-like receptor 2 recognition (Ahn et al. 2018). Here, it was demonstrated that intracellular LTA titers are altered by SPT treatment, but not in a canonical way, as LTA levels decreased at the lower SPT doses in MSSA and abruptly increased in higher doses. In MRSA, the pattern was completely different with increased LTA titers for most of the studied SPT concentrations. Interestingly, secreted LTA could not be detected for MSSA. It is previously reported that MRSA secretes more toxins than susceptible *S. aureus* (Schlievert et al. 2010).

*S. aureus* lacking LTA, or carrying modifications in LTA charge, have altered hydrophobicity and are unable to adhere to cells (Weidenmaier et al. 2004; Fedtke et al. 2007; Naclerio et al. 2020). LTA is directly linked to PGs and, specifically in MRSA, is modified by β-O-linked-*N*-acetyl-glucosaminylations. Inhibiting LTA synthesis or deleting *tarS* - the gene that codes the glycosyltransferase that adds β-*O*-*N*-acetyl-d-glucosamine residues, leads to PGs instability and sensitizes MRSA to β-lactams, thus proving its importance in MRSA phenotype (Campbell et al. 2011; Brown et al. 2012). SPT treatment of MRSA increased LTA extracellular levels dose-dependently, strongly correlating with biofilm inhibition. This effect could indeed imply damage to bacterial cell walls similar to that caused by antibiotics, destabilizing LTA connection and thus leading to its release (van Langevelde et al. 1998; Lotz et al. 2006). This could not be verified in MSSA, which could be due to the lower employed SPT doses studied. Thus, biofilm inhibition could be achieved in an LTA-independent mechanism on those bacteria. Previous studies using LTA from *Lactobacillus plantarum* have shown the biofilm-eradicating ability of LTA against MSSA and MRSA. This did not affect the growth of the bacteria, giving insights into a new role for LTA, possibly participating in the biofilm dispersion phase.

Moreover, LTA was proven to target autolysin A, a crucial molecular player during autolysis (Biswas et al. 2006), which is important for establishing biofilm in its initial steps (Bose et al. 2012). Autolysis is vital for the establishment of *ica*-independent biofilms, as the ones usually found in clinical MRSA strain, especially for the primary attachment steps (Boles et al. 2010; Houston et al. 2011), as well as for the release of extracellular DNA - a crucial factor for EPS stabilization (Bose et al. 2012). On note, SPT was studied here as a biofilm-restrictive agent, which would further underline its possible inhibitory effect in the initial steps of biofilm establishment. Other studies implied that LTA may be involved in osmoprotection (Percy and Gründling 2014) or ion equilibrium (Neuhaus and Baddiley 2003). Thus, the increase of LTA found here could be associated with a stress reaction of the bacteria to the disturbing effect of SPT, blocking autolysis and thus impeding biofilm formation. The differential effect observed between the two studied *S. aureus* strains implies that LTA possesses different roles in the physiology of these bacteria, or that SPT affects MSSA and MRSA cell walls in different ways.

Finally, SPT treatment led to important alterations in phosphate homeostasis, as reflected by the activity of ALPs and levels of DING proteins. ALPs were previously found to be crucial for biofilm formation, as vanadium inhibitors significantly diminished biofilm formation and ALP activity of *S. aureus* in a correlating and dose-dependent manner (Danikowski and Cheng 2018; Katsipis et al. 2021a). In the current study, the extracellular ALP activity was also studied and surprisingly found significantly increased for both MSSA and MRSA, in contrast with its intracellular-localized counterpart(s). This result seems to be complementary to the gradual diminishing effect of SPT doses on intracellular ALP activity and may propose an SPT-induced release of intracellular ALPs to the growth medium. The ALPs of *S. aureus* reside on the surface of the cell wall (Okabayashi et al. 1974), so they may be released from bacteria due to cell wall alterations/damage. A similar pattern for MRSA was established for DING proteins, a group of proteins regulating phosphate binding (Berna et al. 2008), as were found to be increased at the medium by a simultaneous decrease of intracellular levels. ALPs are expressed in biofilm in response to phosphate limitation (Huang et al. 1998). At phosphate starvation, *pho* regulon is upregulated, increasing the titers of ALPs and DING protein to many bacteria to manage low phosphate levels (Zaborina et al. 2008; Santos-Beneit 2015; Vuppada et al. 2018), hindering at the same time biofilm formation (Monds et al. 2001, 2007; Haddad et al. 2009). Subsequently, secretion of phosphate-scavenging proteins, like ALPs and DING, can either digest organophosphates or receive free phosphates, transfer to cell-bound PstS proteins, and serve phosphate nourishment of the bacteria (Berna et al. 2008).

Several virulence factors, including secreted enzymes, are typically produced during the post-exponential and stationary phases of *S. aureus*, especially from MRSA, for the nourishment of bacteria and detoxification processes (Schlievert et al. 2010). Thus, the observations of the study could be attributed to bacterial inability to sustain active metabolism at the latter stages of the culture, including phosphate metabolism and oxidoreductive potential, which will influence many physiological pathways regarding viability and cell wall composition. For instance, *S. aureus* cells deprived of phosphate demonstrate a rapid decrease in viability, becoming non-culturable after 4 days, in parallel with acidification of the culture medium (Watson et al. 1998). In addition, during phosphate limitation, the glycerophosphodiesterase GlpQ of *S. aureus* is expressed and consumes glycerol 3-phosphate (G3P) from wall teichoic acids of neighboring cells and other phospholipids (Jorge et al. 2018). Also, LTA synthesis is blocked under phosphate starvation in *Bacillus subtilis* (Qi et al. 1997; Botella et al. 2014) due to its phosphate-rich nature. *S. aureus* then releases and receives phosphate from G3P, amino acids, nucleotides, and other organic and inorganic phosphoric compounds through an ALP (also known as PhoB)-dependent pathway (Kelliher et al. 2020). In addition, *S. aureus* cells that do not express sortase A do not seem to be able to actively metabolize phosphate, displaying a phosphate starvation profile in the presence of an abundant phosphate source (van der Kooi-Pol 2013), giving an alternate insight into the inhibitory activity of SPT, that reflects the conditions employed in the current study, as adequate phosphate has been introduced to the growth medium. A sortase A dysfunction could also explain the reduction in amyloids found here, and biofilm inhibition as its activity is critical for *S. aureus* surface protein G cleavage and involvement in the accumulation phase of biofilm (Geoghegan et al. 2010).

In summary, SPT is a potent inhibitor for the formation of *S. aureus* biofilm for both MSSA and MRSA, though MSSA is more sensitive to its activity as expected from previous studies with antibacterial agents. SPT is most possibly implicated in metabolic and physiological pathways concerning the homeostasis of phosphorus, the biosynthesis and stabilization of bacterial cell walls, and the processing of amyloid compounds implicated in the initial steps of biofilm formation. These also extend to the restriction of active metabolism, as expressed by viability reduction and the activity of intracellular ALPs. Combating health alert against MRSA with a possible simultaneous SPT therapy should be studied further in the future, as (a) SPT-treated MRSA present destabilized homeostatic and molecular patterns and (b) blocking biofilm formation leads to the accumulation of planktonic bacteria that would be more susceptible to antibiotics and post-exponential phase stress. In the current research, the expression patterns and quantified levels of some crucial molecular factors was not yet studied. More work should be conducted to further prove these notions, implicating their expression, and proteomics protocols, in order to actively implicate SPT in the battlefront against *S. aureus*, especially against multi-drug-resistant strains.

## Supplementary information

ESM 1 (PDF 159 kb)

## Data Availability

The datasets generated during and/or analyzed during the current study are available from the corresponding author on reasonable request.
