# Supplementary material for: Serrapeptase impairs biofilm, wall, and phospho-homeostasis of resistant and susceptible Staphylococcus aureus
Source: Appl Microbiol Biotechnol. 2023 Jan 13;107(4):1373–89. doi: 10.1007/s00253-022-12356-5 (PMC9898353; doi:10.1007/s00253-022-12356-5)
Supplement: Supplementary file 1 — (PDF 159 kb) [file 253_2022_12356_MOESM1_ESM.pdf]

Supplementary Material

Serrapeptase impairs biofilm, wall, and phospho-homeostasis of resistant and susceptible *Staphylococcus aureus*

Applied Microbiology and Biotechnology

G. Katsipis [1], A.A. Pantazaki[*1]

[1] Laboratory of Biochemistry, Department of Chemistry, Aristotle University of Thessaloniki, 54124 Thessaloniki, Greece

***Corresponding author:** Professor A.A. Pantazaki, Lab. of Biochemistry, Dept. of Chemistry, Aristotle University of Thessaloniki, 54124 Thessaloniki, Greece, Tel: +30-2310-997838 & Fax: +30-2310-997689

e-mail: natasa@chem.auth.gr,

Web: https://www.chem.auth.gr/staff/pantazaki/

Inhibition of biofilm formation by SPT

Possible inhibition of biofilm formation by several concentrations of SPT has been studied with two different approaches: i) the tissue culture plate (TCP) method, and ii) the coverslip method, as previously described (Katsipis et al. 2021a).

Bacteria were pre-cultured as described and then diluted 100-times with a TSB medium. For the quantification of biofilm formation, the TCP method was employed. Bacteria were grown in sterile 96-well flat-bottom polystyrene TCPs (#3596 Costar, Corning Inc., Corning, NY, USA), with or without the addition of several SPT concentrations, under biofilm-forming conditions. Wells containing only the growth medium were also employed as negative controls (blanks). The contents of the wells were then discarded, and the wells were washed with 0.1M phosphate-buffered saline solution (PBS), pH 7.2. The plates were dried for 1 h at 50°C and stained with CV (0.1% w/v) for 10 minutes. Then the plates were washed thoroughly with tap water and left to dry for another hour at 50°C. CV stain from biofilms was extracted with 33% acetic acid, and the absorbance was read at 570 nm (A570) in a microplate reader.

For microscopical evaluation of the formed biofilms, bacteria were grown under biofilm conditions, as previously described, in 60 cm^2 Petri dishes, with sterile glass slides fully submerged in the growth medium. After incubation, the glass slides were washed with PBS, heat-fixed, and stained with 0.4 % (w/v) CV for 10 min. The slides were washed thoroughly with tap water and dried for 1 h at 50°C. The stained biofilms on the glass slides were examined under a light microscope at 100X magnification.

The percentage of biofilm inhibition was calculated by using the following formula, after the deduction of blank values:

$$Biofilm\ Inhibition\ (\%) = \frac{Control\ A570 - Treated\ A570}{Control\ A570}\ X\ 100$$

Biofilm formation in the presence of either bovine serum albumin or trypsin

To exclude that the inhibitory effect of SPT is due to its proteolytic activity against the biofilm contributing proteins, the possible inhibitory effect of two proteins, a non-exhibiting and exhibiting protease activity, bovine serum albumin (BSA) and trypsin respectively, was also studied (Figure S1). The presence of BSA did not affect biofilm formation in MRSA bacteria, while trypsin increased biofilm formation at concentrations above 5 μg/mL. Regarding MSSA, only trypsin significantly impaired biofilm formation, but that was demonstrated only for SPT concentrations higher than 5 μg/mL (IC50 = 21.55 μg / mL (CI (95%) = 14.93 - 42.85 μg/mL)). These results prove that SPT possesses a specific anti-biofilm activity.

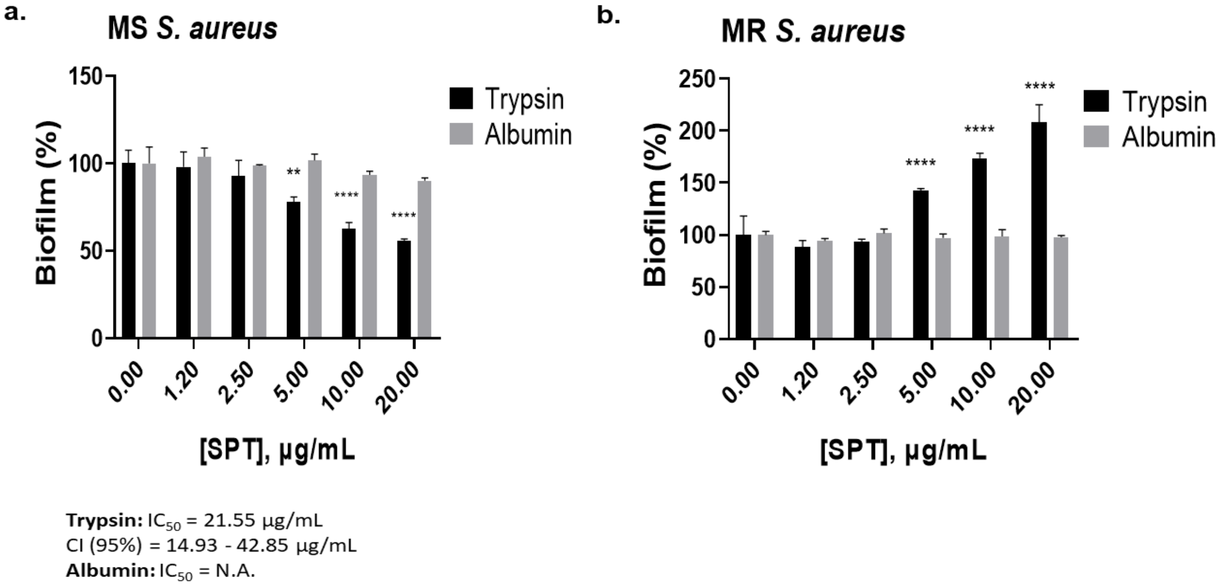

Figure S1 Biofilm formation of **(a)** *S. aureus* ATCC 25922 (Methicillin-susceptible, MSSA), and of **(b)** Methicillin-resistant *S. aureus* (MRSA) ST80, by trypsin (black bars) or albumin from bovine serum (grey bars). Bacteria were grown under stative conditions, in the presence or absence of several concentrations of either trypsin or albumin. Bars represent mean values ± SEM from at least three independent experiments, with the value of the untreated bacteria culture (control) set at 100%. Brown-Forsythe tests, and Welch ANOVA were employed for the statistical analysis. Notations for statistically significant differences between Control (untreated) and treated samples: * $p < 0.05$; ** $p < 0.01$; *** $p < 0.001$; **** $p < 0.0001$.
